# Comparison of supportive regulatory measures for pediatric medical device development in Japan and the United States

**DOI:** 10.1007/s10047-020-01216-6

**Published:** 2020-10-20

**Authors:** Sara Takahashi, Kiyotaka Iwasaki, Haruki Shirato, Mami Ho, Mitsuo Umezu

**Affiliations:** 1 Cooperative Major in Advanced Biomedical Sciences, Joint Graduate School of Tokyo Women’s Medical University and Waseda University, Waseda University, 2-2 Wakamatsucho, Shinjuku, Tokyo, 1628480 Japan; 2 Office of Medical Devices I, Pharmaceuticals and Medical Devices Agency, Shin-Kasumigaseki Building, 3-3-2, Kasumigaseki, Chiyoda-ku, Tokyo, 100-0013 Japan; 3 Department of Modern Mechanical Engineering, Graduate School of Creative Science and Engineering, Waseda University, 2-2 Wakamatsucho, Shinjuku, Tokyo, 1628480 Japan; 4 Department of Integrative Bioscience and Biomedical Engineering, Graduate School of Advanced Science and Engineering, Waseda University, 2-2 Wakamatsucho, Shinjuku, Tokyo, 1628480 Japan

**Keywords:** Pediatric medical device, Regulatory measure, Regulatory science

## Abstract

Further development of medical devices for children is required in Japan, but the development of such devices is delayed compared to that of medical devices for adults. Herein, we investigated policies for advancing the development of pediatric medical devices in Japan and the United States. Considering the achievements of each policy, we proposed a strategy to promote further development of pediatric medical devices in Japan. We investigated policies for supporting the development of pediatric medical devices and approved cases in Japan and the United States by searching contents of websites of regulatory bodies and other related administrations, and scientific papers. We found the main six policies in Japan and nine main policies in the United States for the development of pediatric medical devices. In the United States, various measures have initiated mainly in the 2000s, while in Japan, the main measures have been in place since 2013. Similarities were found in both countries, such as subsidies for application fees and research and development expenses, exemption of requirements for regulatory approval, and priority review and consultation by the regulatory body. Our study revealed that there are similarities in initiatives by both countries. To promote further development of pediatric medical devices in the future, improvements to expediting the review process to approval by the regulatory body, global development, and implementation of alternative measures to ensure the efficacy and safety of the device instead of large-scale clinical trials should be anticipated through cooperation among industry, government, and academia.

## Introduction

In Japan, the birthrate is declining, and further development of medical devices for children is needed. It is often difficult for companies to dedicate efforts on the development of pediatric medical devices because of the small number of patients in the pediatric field, complicated disease conditions, difficulties in assessing efficacy and safety of pediatric medical devices, and the necessity of multiple device variations.

As for medical devices for adults, pediatric medical devices are categorized into four classes (classes I to IV) depending on their risk levels according to the “Global Harmonization Task Force (GHTF)” rule. For class II devices, no review standard exists, while for medium-risk (class III), and high-risk (class IV) medical devices, a marketing holder requires marketing approval from the Minister of Health, Labour and Welfare (MHLW). Prior to the MHLW granting approval to an industry for each medical device, the office of review of medical devices I and II at the Pharmaceutical and Medical Devices Agency (PMDA) reviews the efficacy and safety of each medical device scientifically [1]. Medical devices reviewed by the PMDA are classified as “Brand-new,” “Improved,” and “Me-too” medical devices. Brand-new medical devices are defined as those that have a novel structure, usage, indication, or performance compared to other medical devices approved in Japan regardless of their class. In the United States, the Center for Devices and Radiological Health in the Food and Drug Administration (FDA) is responsible for the review of all medical devices [2].

In Japan, an adult is defined as aged 20 years or older by the Japanese Civil Code, while in the United States, an adult is defined as aged 22 years or older according to the Federal Food, Drug, and Cosmetic Act (FD & C Act) and medical devices used for patients under the age of 21 are recognized as pediatric medical devices. Thus, the definition of age for pediatric medical devices differs between the two countries. Moreover, for congenital diseases, treatment may continue as under-aged but also throughout adulthood; thus, it is difficult to delineate the definition by age. In this study, a medical device targeted for the treatment and diagnosis of under-aged, but also a medical device targeted for the treatment and diagnosis of congenital diseases is referred to as a “pediatric medical device”.

In an attempt to promote timely development and regulatory approval of pediatric medical devices, we investigated and compared policies for advancing the development of pediatric medical devices in Japan and the United States since the regulation of medical devices in both countries is similar. Considering the achievements by policies in each country, we propose a strategy to be implemented for encouraging the development of pediatric medical devices in Japan.

## Materials and methods

**Fig. 1:**
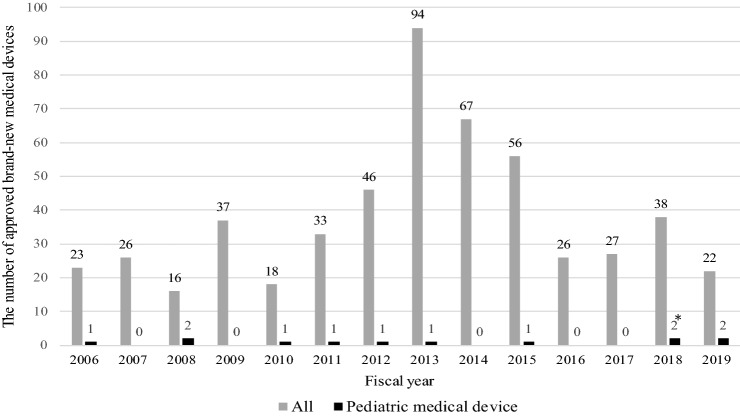
The number of approved brand-new medical devices in Japan from April 2006 to December 2019. *2 pediatric medical devices approved in 2018 are used for blocking blood flow to the acardiac fetus

According to the PMDA website, 529 brand-new medical devices were approved from April 2006 to December 2019 [3]. The number of medical devices used for the treatment of the pediatric population diseases or congenital diseases, defined as “pediatric medical devices” in this study, is 12 (12/529, 2.3%). Figure 1 shows the number of approved brand-new medical devices in each fiscal year from 2006 to 2019. The number of approved brand-new pediatric medical devices in each fiscal year is 0–2, which is very few compared to the number of medical devices for the adult population. In this study, we investigated policies for supporting the development of pediatric medical devices and approved cases in Japan and the United States by searching contents of websites of the PMDA, the MHLW, the FDA, other related administrations, and scientific papers.

### Policies in Japan

Japanese policies for medicine and medical devices are available on the website of the MHLW [4]. Based on the available information released on the website of the MHLW as of December 2019, three policies are assumed to be strongly associated with the promotion of medical device development; "*Study Group on the Early Introduction of Medical Devices, *etc*. with High Medical Need*" [5], "*Orphan Medical Device Designation System*” [6], and “*SAKIGAKE Designation System*” [7]. In addition, a search for “pediatric medical devices” and “medical device, early approval” on the PMDA website [8] by December 2019 revealed three unique initiatives in Japan that are expected to be strongly related to the promotion of the development of pediatric medical devices; “*Conditional Early Approval System for Innovative Medical Device Products*” [9], “*PMDA Science Board on Evaluation of Medical device in Pediatric Use*” [10] and “*Subsidization program for application of pediatric medical device*” [11]. Thus, we investigated these six Japanese policies. We searched the purpose and achievement of each initiative based on the published information by the MHLW and the PMDA. Regarding the *Orphan Medical Device Designation System*, we also referred to the web information provided by the Japan Association for the Advancement of Medical Equipment (JAAME) [12]. Moreover, we analyzed the utilization of each policy in terms of regulatory approval using the PMDA’s review reports of the approved pediatric medical devices. With regard to individual products, we also referred to the materials released by companies.

### Policies in the United States

To investigate the policies in the United States, we performed a web-search on PubMed on August 11, 2019 using the search formula "Pediatric AND “medical device*” AND Regulation". We found 69 papers by the search, and we examined four papers summarizing regulation of pediatric medical device in the United States [13–16]. Furthermore, we investigated the policies and initiatives implemented through the FDA's Web pages for “Pediatrics” [17], “Pediatric Medical Devices” [18], and “Public meeting—Pediatric Medical Device Development” ^[19]^, published by December 2019. As a result, we found nine initiatives related to the promotion of the development of pediatric medical devices. We investigated the purpose of each policy and the status of utilization for approval of pediatric medical devices based on public information including the FDA website.

## Results

### Efforts and achievements in Japan

Table 1 shows the purposes and achievements of each measure in Japan. As shown in the purpose column in Table 1, only the sixth “*Subsidization program for application of pediatric medical device*” is specialized for pediatric medical devices. Other initiatives were established for medical devices for rare diseases and very innovative medical devices for disease with no other effective treatment. Five policies started in 2013 or later in Japan. We found 16 (16/140, 11.4%) of the adopted devices as the “*Study Group on the Early Introduction of Medical Devices, *etc*. with High Medical Need*” were pediatric medical devices, by the materials of the 29th meeting of "Study Group on the Early Introduction of Medical Devices, etc. with High Medical Need" in October 2018 [20], and 7 of the 16 (7/16, 43.8%) pediatric devices were approved following designation by the study group. Three (3/30, 10.0%) pediatric medical devices were selected as the target of the “*Orphan Medical Device Designation System*” and, all (3/3, 100.0%) pediatric medical devices with “*Orphan Medical Device Designation System*” have been approved for use [12]. By the 2019 fiscal year, one (1/9, 1.1%) pediatric medical device was selected as the target of the “*SAKIGAKE Designation System*”, which began in 2015 [21]. As for the pediatric medical device designated in the “*SAKIGAKE Designation System*”, a domestic clinical trial for the pediatric medical device has started to obtain evidence for regulatory approval [22]. Furthermore, “*the PMDA Science Board on Evaluation of Medical device in Pediatric Use”* which was commissioned by the PMDA started a discussion related to the development delay of pediatric medical devices, and a summary report was issued in 2015 [23]. In addition, the “*Conditional Early Approval System for Innovative Medical Device Products*” was launched in 2017 [9, 24]. Although no medical device has been approved using the scheme as of the 2019 fiscal year, an application for approval of the medical device for the treatment of congenital heart disease using the “*Conditional Early Approval System for Innovative Medical Device Products*” was mentioned in the 29th meeting of "Study Group on the Early Introduction of Medical Devices, etc. with High Medical Need" in October 2018 [20, 25]. “*Conditional Early Approval System for Innovative Medical Device Products*” and “*SAKIGAKE Designation System*” were regulatory approaches, however, the Japanese act, “the Act on Securing Quality, Efficacy and Safety of Pharmaceuticals, Medical Devices, Regenerative and Cellular Therapy Products, Gene Therapy Products, and Cosmetics”, was approved to be amended in 2019, and these policies were subsequently enacted formally [26].

**Table 1:** Supportive regulatory measures for pediatric medical device development in Japan

	Name	Purpose	Incentives to developers	Inaugural year	Results
1	Study Group on the Early Introduction of Medical Devices, etc. with High Medical Need	Assessing the medical necessity of submitted requests for development and promoted development by companies by selecting those with high medical need in Japan	・Priority review ・Subsidy reimbursement	2006	140 medical devices had been designated by the 29th meeting in October 2018 and 16 (16/140, 11.4%) pediatric medical devices were included 7 of the 16 (7/16, 43.8%) pediatric devices were approved following designation by the study group
2	Orphan Medical Device Designation System	Supporting and promoting research activities for the development of orphan medical devices	・Priority review ・Subsidy payment ・Guidance and consultation with regulatory authorities ・Preferential tax treatment ・Extension of re-examination period	2013	30 medical products were designated by December 2019 and 3 (3/30, 10.0%) pediatric medical devices were included. All 3 3 (3/3, 100.0%) pediatric medical devices were approved after designated
3	PMDA Science board on Evaluation of Medical device in Pediatric Use	Advancing regulatory science and evaluate products with advanced science and technology in appropriate manner by enhancing cooperation and communication with academia and medical institutions, based on PMDA's philosophy to deliver safe and effective drugs and medical devices to the people and further promotion of medical innovations	・Providing consideration on the evaluation of efficacy and safety of medical device for pediatric population	2014 (until 2015)	The report was published by PMDA Science Board on Evaluation of Medical device in Pediatric Use
4	SAKIGAKE Designation System	Promoting R&D in Japan aiming at early practical application for innovative medical devices	・Offering a concierge service ・Prioritized Consultation ・Priority review ・Subsidy reimbursement	2015	9 medical devices were designated by FY2019 and 1 (1/9, 1.1%) pediatric medical device was included. A clinical trial for evaluating the pediatric medical device is ongoing
5	Conditional Early Approval System for Innovative Medical Device Products	Accelerating patient access to innovative medical devices intended to treat life-threatening diseases for which no therapies whose benefits outweigh the risks currently exist	・Priority review ・Allowing companies to apply pre-marketing submission with limited clinical data instead of clinical trials conducted under the Ministerial Ordinance on Good Clinical Practice for Medical Devices	2017	Although no medical device has been approved using the scheme as of the 2019 fiscal year, an application for approval of medical device for the treatment of congenital heart disease using the measure was mentioned in the 29th meeting of "Study Group on the Early Introduction of Medical Devices, etc. with High Medical Need" in October 2018
6	Subsidization program for application of pediatric medical device	Supporting and promoting development of pediatric medical devices	・Subsidy payment	2019	No medical device has been designated

### Efforts and achievements in the United States

Table 2 shows the efforts and achievements for accelerating the development of orphan medical devices and very innovative medical devices in the United States. The FDA established “*Office of Orphan Products Development*” [27] and “*Office of Pediatric Therapeutics*” [28] to ensure access to innovative, safe, and effective medical products for the treatment of orphan diseases and pediatric population diseases. Approved pediatric medical devices were reported annually in the *“Pediatric Report to Congress”* [29–35], indicating that the percentage of approved pediatric medical devices has remained approximately 10–20% of the total number approved. In the United States, a program to support clinical trials of orphan products, “*Orphan Products Clinical Trials Grants Program*”, began in 1983 [36]. The “*Pediatric Advisory Committee*”, a third-party agency of the FDA, was launched in 1999 [37], and 5 FDA guidelines for the development of pediatric medical devices were issued from 2003 to 2016 [38–42]. The “*Pediatric Medical Device Safety and Improvement Act*” was enacted to promote the development of pediatric medical devices in 2007. The law promotes the following six themes and many initiatives defined by the law: “*tracking pediatric device approvals”,* “*modification to humanitarian device exemption”,* “*encouraging pediatric medical device research”,* “*device development consortia”,* “*pediatric advisory committee”,* and “*post-market surveillance”* [43]. In addition, the “*Humanitarian Use Device” (HUD)/”Humanitarian Device Exemption” (HDE)*, which is an approval pathway for medical devices directed at fewer than 8000 patients a year, has been restructured with no upper limit on profits for an industry who develops pediatric medical devices [14, 44]. The FDA approved 100 medical devices out of total 418 approved medical devices, indicated for use in a pediatric population or subpopulation, and nine of 100 (9/100, 9.0%) pediatric medical devices were approved using “*the Humanitarian Device Exemption (HDE) system”* from the fiscal year 2009–2017 [29–35]. *The Pediatric Device Consortia Grant Program*, which is derived from *the “Pediatric Medical Device Safety and Improvement Act”*, provided approximately $3.5–$6 million annually to select the Pediatric Device Consortia, and 19 medical devices have been approved as of September 2018 [45, 46]. Furthermore, the “*Breakthrough Device Program*”, replaced the “*Expedited Access Pathway Program*” in 2017, specifies regulatory measures for more effective treatment or diagnosis of life-threatening or irreversibly debilitating diseases or conditions [47]. FDA selected ninety-seven medical devices as the “*Breakthrough Devices Program*” or *Expedited Access Pathway Program* as of September 30, 2018*.* 24 (24/97, 24.7%) investigational device exemptions (IDEs) have been approved or completed for clinical studies of breakthrough devices, and nine (9/97, 9.3%) breakthrough devices have had a marketing submission approved, cleared, or granted as of September 30, 2018 [48]. In addition, the “FDA Public Meeting; Pediatric Medical Device Development” was held in 2018 and stakeholders from academia, industry and the FDA exchanged views on accelerating medical device innovation [19].

**Table 2:** Supportive regulatory measures for pediatric medical device development in the United States

	Name	Purpose	Incentives to developers	Inaugural year	Results
1	Orphan Products Clinical Trials Grants Program	Supporting clinical trial research of the development of new safe and effective medical products for rare diseases/conditions	・Subsidy payment	1983	At any one time, there are typically 60 to 85 ongoing grant-funded projects. A major portion of the appropriated funds (typically approximately $15.5 million) for a given fiscal year go towards continued funding of prior approved grants
2	Pediatric advisory committee	Advising and making recommendations to the Commissioner of Food and Drugs regarding pediatric research, clinical trials, pediatric labeling, adverse event reports		1999	Meetings are held every year and three meetings were held in 2018
3	FDA guidance related development of pediatric medical devices	Representing the FDA's current thinking on each topic to industries and FDA Staffs	・Providing consideration on the evaluation of efficacy and safety of medical device for pediatric population	2003	・Pediatric Expertise for Advisory Panels (2003) ・Premarket Assessment of Pediatric Medical Devices (2014) (This document supersedes “Guidance for Industry and FDA Staff: Premarket Assessment of Pediatric Medical Devices” dated May 14, 2004.) ・Providing Information about Pediatric Uses of Medical Devices (2014) ・Leveraging Existing Clinical Data for Extrapolation to Pediatric Uses of Medical Devices (2016) ・Pediatric Information for X-ray Imaging Device Premarket Notifications (2017)
4	Pediatric Medical Device Safety and Improvement Act (PMDSIA)	Creating new incentives, mandates, FDA authority and funding with the aim of increasing the availability of devices for pediatric populations while assuring the safety and effectiveness of those devices	・Improving the environment for supporting development of pediatric medical devices	2007	Many programs for supporting development of pediatric medical devices started by the Act
5	Humanitarian Use Device (HUD)/Humanitarian Device Exemption (HDE)	A medical device intended to benefit patients in the treatment or diagnosis of a disease or condition that affects or is manifested in not more than 8,000 individuals in the United States per year/a marketing application for Humanitarian Use Device (HUD)	・Subsidy payment ・Approved with probably benefit	2008 (Revised HUD/HDE)	The FDA approved 100 medical devices out of total 418 approved medical devices, indicated for use in a pediatric population or subpopulation, and nine of 100 (9/100, 9.0%) pediatric medical devices were approved using “the Humanitarian Device Exemption (HDE) system” from the fiscal year 2009 to 2017
6	Reports to Congress	Fiscal year report from FDA about approved pediatric medical devices	・Providing information about approved pediatric medical devices	2008	Approved pediatric medical devices were reported annually in the Pediatric Report to Congress
7	Pediatric Device Consortia (PDC) Grant Program	Supporting the development of nonprofit consortia designed to stimulate projects which will promote pediatric device development	・Subsidy payment	2009	The program provided approximately $3.5 to $6 million annually to select the Pediatric Device Consortia since 2009
8	Breakthrough Device Program (Expedited Access Pathway)	Expediting the development and prioritize the review of certain medical devices that provide for more effective treatment or diagnosis of life-threatening or irreversibly debilitating diseases or conditions	・Priority review ・Expedited Access Pathway	2017 (2015)	FDA selected ninety-seven medical devices as the “Breakthrough Devices Program” or Expedited Access Pathway Program as of September 30, 2018. 24 (24/97, 24.7%) investigational device exemptions (IDEs) have been approved or completed for clinical studies of breakthrough devices, and nine (9/97, 9.3%) breakthrough devices have had a marketing submission approved, cleared, or granted as of September 30, 2018
9	FDA Public Meeting; Pediatric Medical Device Development	Identifying strategies that enhance the medical device ecosystem toward development and innovation of devices that serve the complex needs of children, and thereby accelerating medical device innovation for all Americans	・Exchanging views among industry, academia and regulatory body	2018	FDA Public Meeting regarding pediatric medical device development was held in 2018

## Discussion

### Comparison between Japan and the United States

**Fig. 2:**
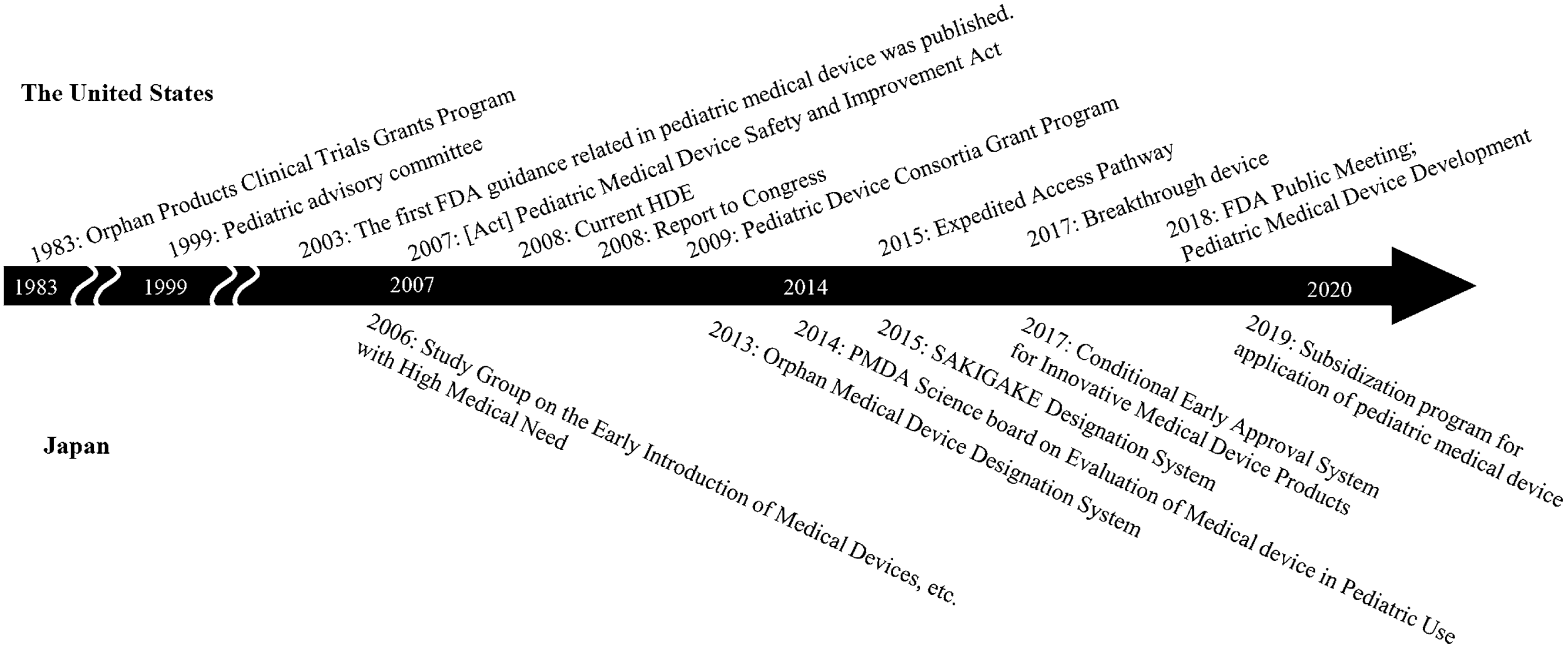
History of supportive regulatory measures for pediatric medical device development in Japan and the United States

Table 3 and Figure 2 show a comparison of the measures present in Japan and the United States. In the United States, various measures have been in effect mainly since the 2000s, while in Japan, the main measures have been available since 2013. Our analysis shows that similarities are found in both countries, such as subsidies for application fees and research and development expenses, exemption requirements for regulatory approval, and priority review and consultation by the regulatory body. In Japan, a specialized consortium has not been established, and the budget for pediatric medical devices is small compared to that of the United States. However, it has been confirmed that a priority review and consultation by PMDA contributes to expediting the review process to the approval of such devices in Japan.

**Table 3:** Similarities and differences between actions for pediatric medical device development in Japan and the United States

	Similar actions	Main measures	
		Japan	The United States
1	Review of medical devices in regulatory authority	・Office of review of medical device I and II in PMDA	・Center for Devices and Radiological Health (CDRH) in FDA
2	Guidance for developers	・PMDA Science board report	・FDA guidance
3	Grant program for development of pediatric medical devices	・Orphan Medical Device Designation System	・Pediatric Device Consortia (PDC) Grant Program ・Orphan Products Clinical Trials Grants Program
4	Exempted requirements for approval	・Conditional Early Approval System for Innovative Medical Device Products	・Humanitarian Use Device(HUD)/Humanitarian Device Exemption (HDE)
5	Subsidy payment for application and consultation with regulatory authority	・Orphan Medical Device Designation System ・Subsidization program for application of pediatric medical device	・Humanitarian Use Device(HUD)/Humanitarian Device Exemption (HDE)
6	Priority review	・Study Group on the Early Introduction of Medical Devices, etc. with High Medical Need ・Orphan Medical Device Designation System ・SAKIGAKE Designation System ・Conditional Early Approval System for Innovative Medical Device Products	・Humanitarian Use Device(HUD)/Humanitarian Device Exemption (HDE) ・Breakthrough Device Program
7	Expedited Access Pathway/ Prioritized Consultation	・SAKIGAKE Designation System	・Breakthrough Device Program

	Different actions	Main measures	
		Japan	The United States
1	Specialized act for development of pediatric medical devices	・Conditional Early Approval System for Innovative Medical Device Products* ・SAKIGAKE Designation System*	・Pediatric Medical Device Safety and Improvement Act
2	FDA office for supporting pediatric medical device		・Office of Pediatric Therapeutics ・Office of Orphan Products Development established in FDA
3	Independent committee for pediatric medical devices		・Pediatric advisory committee
4	Specialized consortium for pediatric medical device development		・Pediatric Device Consortia (PDC) Grant Program
5	Meeting for stakeholders from industry, academia and regulatory		・FDA Public Meeting; Pediatric Medical Device Development

*Japanese act, “the Act on Securing Quality, Efficacy and Safety of Pharmaceuticals, Medical Devices, Regenerative and Cellular Therapy Products, Gene Therapy Products, and Cosmetics”, was approved to be amended in 2019, and these policies were subsequently enacted formally

### What is the effective solution for the further development of pediatric medical devices?

Although both countries began to focus on promoting the development of pediatric medical devices at different times, various efforts are being undertaken in Japan and the United States, and these initiatives are similar in both countries. In some cases, these measures have led to approvals of pediatric medical devices. Despite these efforts, the number of approvals for pediatric medical devices in both the United States and Japan has been stagnating. For the future development of pediatric medical devices, we considered a potential strategy to be implemented in Japan.

Throughout our study, we considered that the following three issues should be prioritized for further development of pediatric medical devices: (1) contribution to expediting the review process by the approval by regulatory body, (2) support of global development, and (3) implementation of an alternative evaluation method in lieu of a large-scale clinical trial. We found the FDA and the PMDA have contributed priority review and consultation for various measures and these initiatives have led to the approvals of medical devices. Contribution by the regulatory body is considered to be supportive for innovators. Recently, a collaboration between the FDA and the PMDA, known as the “*Harmonization By Doing*” and “Collaboration Review”, has begun [49–52]. Global collaboration is a significant issue for the development of pediatric medical devices since the number of target patients is small in each country. Although the requirements in the review process for regulatory approvals in each country are sometimes different, the FDA and the PMDA have focused on reducing pre-marketing requirements by making pre- and post-marketing evaluations more accessible and appropriate. In clinical trials of high-risk innovative medical devices, high costs are a notable hurdle, especially in pediatric medical devices. The “*Conditional Early Approval System for Innovative Medical Device Products*” policy in Japan allows companies to apply pre-marketing submission with limited clinical data instead of clinical trials conducted under the Ministerial Ordinance on Good Clinical Practice for Medical Devices [53], and it is expected to be an option for early application for pediatric medical devices. Under this system, in order for a medical device to be safely introduced in Japan, it is necessary to formulate adequate standards for users and facilities, collaborate with academic societies, conduct post-marketing surveillance, and gradually expand the number of facilities. Cooperation among industry, government, and academia is prerequisite for expediting the approval of new pediatric medical devices based on such limited data. When the *Pediatric Medical Device Safety and Improvement Act* was enacted in the United States, “American Academy of Pediatrics” held a meeting with medical societies, advocacy groups, industry, researchers, and government officials to identify and remediate barriers to pediatric device development [14]. The opportunities for discussion among stakeholders in various fields, including public meetings held by the regulatory bodies and the “Harmonization-by-Doing”-children program [54], are expected to be expanded.

Furthermore, it is important to create a mechanism for conducting clinical trials efficiently and to propose a method for evaluating medical devices instead of conducting clinical trials. To allow the conduction of clinical trials in a short term, the “Japanese Society of Pediatric Cardiology and Cardiac Surgery” provides support program, which involves assistance on planning a trial, patient registration, and report-writing [55]. In the development of pediatric medical devices, it is difficult to collect a sufficiently large number of cases and register these cases in clinical trials due to the small number of target patients and the anatomical complexity of patients. We think it is important to use non-clinical evaluation approach rather than clinical trials. For example, when the “EXCOR Pediatric Ventricular Assist Device” was presented, non-clinical tests were conducted to evaluate air leaks in the driving tube, which had been reported in overseas clinical sites [56]. In the review of AMPLATZER Piccolo Occluder, an evaluation based on the shapes of patent ductus arteriosus (PDA) was performed with reference to limited clinical trial data [57]. Although the number of target patients for the AMPLATZER Piccolo Occluder was small in Japan, there were six types of PDA shapes to be taken into consideration. It is often difficult to adequately evaluate device malfunction and performance for an individual patient's anatomical shape and condition using only small-scale clinical trial data. If the evaluation by clinically-relevant non-clinical models is conducted easily and expanded, the efficacy and safety of each pediatric medical device is expected to be better guaranteed for complicated anatomies.

The insurance reimbursement is one of the significant issues for promoting the development of pediatric medical devices. In Japan, industries can apply for a premium pricing category for medical devices which are developed for pediatric disease and for medical devices selected in the “Study Group on the Early Introduction of Medical Devices, etc. with High Medical Need” or the “Orphan Medical Device Designation System”. For instance, the Abbott get the 20% additional pricing for “AMPLATZER Piccolo Occluder” [58]. We consider that it is also important to utilize regulatory policies which we described in this study in terms of obtaining high insurance reimbursement.

## Limitations

There are some limitations to our study. First, we used only published information. Second, we investigated only regulatory measures in Japan and the United States. Nevertheless, the method presented here is useful to discuss future strategies to promote the development and regulatory approval of pediatric medical devices in Japan.

## Conclusion

Policies for supporting the development of pediatric medical devices and the achievements in Japan and the United States were thoroughly investigated. Our study revealed that there are similarities among the initiatives between both countries, such as subsidies for application fees and research and development expenses, exempting requirements for regulatory approval, and priority review and consultation. To promote further development of pediatric medical devices, we considered that improvements to expediting the review process to the approval by a regulatory body, support of global development, and implementation of alternative measures to ensure the efficacy and safety of the device in lieu of large-scale clinical trials will be needed through stimulating better cooperation among industry, government, and academia. Especially for pediatric medical device, future research on the evolution of clinically-relevant device-specific in vitro test method may promote timely development and regulatory approval.
